# Doctor's Precaution against Premature Burial

**Published:** 1914-05-02

**Authors:** Jas. R. Williamson

**Affiliations:** 100 Chedington Road, Upper Edmonton, N.


*To the Editor of* The Hospital.

### Doctor's Precaution Against Premature Burial.

*To the Editor of* The Hospital.

Sir,—Referring to the interesting report under the above title in your excellent Journal of April 18, may I be allowed to point out that a short time since a well-known Metropolitan daily published the narrow escape of a Cossack, named Chourtenko, from interment while alive? It appears that the man's death was duly certified by the Russian authorities at Karpovshaja, near Tsaritzin, and after the body had lain in the morgue for two days it was buried. The coffin had been lowered to the grave and the usual spadeful of earth cast on it by the mourners, when repeated knocking and muffled cries were heard proceeding from the grave. The majority of those present fled in terror, but some of the nearest relatives mustered up courage to descend into the grave and bring the coffin to the surface where it was opened. The medically certified dead man then stated that for some days he had lost the power of speech, but was able to hear, and so was conscious the whole time of the preparations for his funeral and interment. Chourtenko was carried back triumphantly to the village, where he had the extraordinary experience of partaking of his own funeral feast, which, in the customary style, was spread at the house of the military governor. The two doctors who attended the Cossack in his illness were also present!

It was a similar occurrence to the above, of which he was an eye-witness (where a young lady showed signs of life by screaming, and was rescued when the first clods of earth were thrown on her coffin during burial), that led Count Karnicè Karnicki, Chamberlain to the Czar of Russia, to invent his mechanical contrivance for the rescue of people who may have the misfortune to be buried alive. If any of your readers desire further (printed) information on the tragedy of interment alive and its prevention, I shall be happy to send it free of cost on receipt of an envelope stamped and addressed. Thanking you in anticipation of your customary courtesy and kindness,—I am, etc.,

Jas. R. Williamson.

100 Chedington Road, Upper Edmonton, N.

